# Occupational Safety Climate and Hazards in the Industrial Sector: Gender Differences Perspective, Saudi Arabia

**DOI:** 10.3389/fpubh.2022.873498

**Published:** 2022-05-26

**Authors:** Norah AlMousa, Nadin Althabet, Sarah AlSultan, Faisal Albagmi, Heba AlNujaidi, Khaled F. Salama

**Affiliations:** ^1^Department of Public Health, College of Public Health, Imam Abdulrahman Bin Faisal University, Dammam, Saudi Arabia; ^2^Department of Physical Therapy, College of Applied Medical Sciences, Imam Abdulrahman Bin Faisal University, Dammam, Saudi Arabia; ^3^Environmental Health Department, College of Public Health, Imam Abdulrahman Bin Faisal University, Dammam, Saudi Arabia

**Keywords:** gender differences, safety climate, women health, Saudi Arabia, hazards

## Abstract

**Background:**

Occupational Health and Safety (OHS) has become a growing public health concern worldwide. A considerable body of literature has been generated around the theme of safety climate perceptions and occupational hazards, as many researchers have examined perceptions of the occupational safety climate in relation to organizational hierarchy. But there is an urgent need to address safety problems associated with gender differences, especially in Saudi Arabia, where women have only recently begun to work in the industrial field. Therefore, this study aims to assess workers' perceptions of the safety climate and OHS hazards and identify gender differences among workers.

**Methods:**

A cross-sectional study was carried out, using the Nordic Occupational Safety Climate Questionnaire (NOSACQ-50) to assess seven dimensions of safety climate perception and a standardized structured questionnaire adapted from the National Institute of Occupational Health to assess occupational hazards.

**Results:**

The results indicated that respondents perceived only one dimension of their relevant occupational safety climates positively. “Peer safety communication, learning, and trust in co-workers”. Workers experienced a range of different occupational hazards in factories, with noise being the most common. There were significant differences (<0.05) between females and males in the areas of safety priority and risk non-acceptance. Women were more likely to experience ergonomic problems than their male counterparts.

**Conclusion:**

The present study concludes that industries need to comply with national and international OHS standards and rules, especially related to gender perspectives and hazards, as well as provide proper occupational health services in their factories.

## Introduction

OHS is considered integral to public health due to the significant effect it has on the work environment. The primary purpose of OHS is to prevent work-related illnesses, diseases, injuries, deaths, and all other forms of bodily harm that could result from work-related activities (1).

In the 1980s, two measurable concepts, “safety culture” and “safety climate,” were developed. These terms originated from broad notions of organizational culture and organizational climate (2). Zohar developed the first study to evaluate safety climate using a 40-item questionnaire that was distributed to 20 factories (3). The 1990s experienced an increase in the number of publications on safety culture and safety climate and the development of new tools to measure these concepts (3).

Positive motivation toward a safety climate for employees showed a high correlation with safety behavior and health outcomes (4, 5). In a recent cross-sectional study, Ajslev et al. investigated the safety climate and work accidents among 15,000 general working populations (6). They found that young people experienced more work accidents and safety climate challenges. Additionally, the study indicated that lower safety climate scores correlated with a higher number of workplace accidents. Overall, research has shown that occupational accidents and safety climate problems are critical issues in all industries. The published literature support the notion that safety climate perceptions can predict occupational accidents (7).

The literature reveals that the type of work-related injuries and illnesses characteristic and patterns tends to differ based on gender. For example, a study in the electric power industry found that males have a greater risk of injuries than females. However, females had higher injury rates than men in selected occupations, such as that of meter reader (8). A study of the footwear industry found that male and female workers have significant OHS differences in several aspects. For instance, female industrial workers experienced higher psychological stress, had less time to be physically active, and a higher chance of developing musculoskeletal disorders than their male counterparts (9). However, there is limited research conducted to assess women's role in occupational health and safety in industries. Therefore, this study aims to assess the perception of safety climate and occupational hazards and identify gender differences among workers surveyed.

## Materials and Methods

### Research Design and Study Setting

A descriptive cross-sectional study was carried out to identify and measure common occupational hazards in the workplace, the OHS services provided, and workers' perceptions of the safety climate. A structured modified questionnaire was used, and the data were collected using an online survey. The study was conducted in five different factories targeting employees of both genders, including workers, supervisors, and factory managers. We used Marín et al. as reference on methodology duo to the topic similarity.The sample size was calculated using the formula [sample size], where z is confidence level at 95% (standard value of 1.96), is the standard deviation, and the acceptable error = 0.05 (11). Based on this calculation, a minimum sample size of 234 is needed. However, the study collected only 111 responses who accepted to participate in the research study from five different factories. Both male and female participants were required to meet the following inclusion criteria. They needed to be between 18 and 59 years old and able to speak Arabic. Subjects who did not meet these requirements were excluded from the study.

### Sampling Selection and Procedure

The data were collected based on the categorization of Saudi authority for industrial cities and technology Zones (MODON) by the level of industry risk, which is low, moderate, and high-risk industries. One of the inclusion criteria was to select industries with workers from both genders. The online questionnaire administrated by three tablets on the site of each industry. After the participate responded agreed via oral informed consent, each researcher fills the questionnaire through a tablet by interview each worker and ask them for 10–15 min.

### Data Collection Tools

To assess occupational hazards, the standardized structured questionnaire was adapted from surveys issued by the Indian Association of Occupational Health and National Institute of Occupational Health, which were modified to suit the objectives of this study (10). A Theoretical framework of the Nordic Occupational Safety Climate Questionnaire (NOSACQ-50) was used to assess the factories safety climates (11). The survey responses were measured on a Likert scale 1 – 5 where, 1 = highly disagree, 2 = disagree, 3 = neither agree or disagree, 4 = agree, and 5 = highly agree.

The resulting sociodemographic and occupational hazards questionnaire was translated into Arabic by the researchers. A translation validity test was conducted to ensure that the translation was correct and accurate. Face and content validity were conducted to ensure the questionnaire was valid in this context. Furthermore, this questionnaire was organized according to a specific template to ensure validity. The study has been reviewed and approved by the Institutional Review Board [IRB - UGS-2020-03-028] at Imam Abdulrahman Bin Faisal University.

### Statistical Analysis

All analyses were carried out using SPSS, version 20. Descriptive statistical methods were used to describe the frequency and percentage of workers' demographics and individual characteristics and to identify common workplace hazards reported. Industrial workers' perceptions of safety climate were analyzed following the NOSACQ-50 guidelines for each respondent, by which the true mean for each dimension was estimated. The reliability test was calculated using Cronbach's alpha for each safety climate dimension and the chi-square test was used to determine whether there was an association between gender and safety climate perceptions. Moreover, Mann Whitney was employed to determine whether there was statistical evidence that the associated gender means were significantly different. For all variables, the alpha value of 0.05 was considered significant.

## Results

### Descriptive Statistics

Data was collected from 111 participants who responded to the questionnaire. Study respondents included managers, supervisors, and workers from five factory categories, one high-risk chemical and oils factory, one medium-risk plastic rope factory, and three low-risk food factories.

The factory profiles are presented in Table 1. The administrators of all five factories were asked if there is a person responsible for occupational health and safety in their factory. Four of the factories had occupational health and safety specialists, and only one factory did not have someone responsible for OHS. Also, they were asked if they provided an ambulance service; only the high-risk factory provided an ambulance service.

**Table 1:** Factory profile of occupational health and safety services provided to the workers in the factories.

		**Person responsible**
		**for occupational**	**Ambulance**
**Factory activity**	**Factory type**	**health and safety**	**services**
Chemicals and oils	High risk	Yes	Yes
Plastic rope	Medium risk	No	No
Food 1	Low risk	Yes	No
Food 2	Low risk	Yes	No
Food 3	Low risk	Yes	No

### Demographics Characteristics

Demographics characteristics are defined by frequency and parentage. From a total of 111 participants, 22.5% of respondents were from a high-risk category, 26.1% were medium-risk and the 51.4% were low-risk. Participants' ages ranged from 18 to 54 years; 28.8% were aged between 30 and 34. Females represented 57.7% of total participants, and the remaining 43.3% were male; 24.3% were non-Saudi, while 75.7% were Saudi. Married respondents made up 55.9% of the sample; 39.6% of participants had college degrees; 5.4% had diplomas; 43.2% had completed high school; 8.1% had intermediate education; and 3.6% had elementary education. Managers and supervisors made up 25.2% of the participants, while the remaining 74.8% were factory workers. Of all participants, 82% reported they did not smoke, compared to 8% who said they did. Respondents were also queried about the total number of working years that they had been employed by their relevant companies, to which 12.6% replied they had worked for <1 year, 20.7% had worked for 1–3 years, and 26.1% had worked for 4–6 years. Of the remaining respondents, 29.7% reported that they had worked for 7–9 years, while 10.8% of participants had worked for 10 years or more. These results are reflected in Table 2.

**Table 2:** Descriptive analysis of the demographics characteristics.

**Demographic variables**	**Frequency**	**Percentage (%)**
**Factory category**		
High-risk	25	22.5%
Medium risk	29	26.1%
Low risk	57	51.4%
**Age**		
18–24	7	6.3%
25–29	28	25.2%
30–34	32	28.8%
35–39	29	26.1%
40–44	10	9.0%
45–49	3	2.7%
50–54	2	1.8%
**Gender**		
Female	64	57.7%
Male	47	42.3%
**Nationality**		
Non–Saudi	27	24.3%
Saudi	84	75.7%
**Marital status**		
Divorced	3	2.7%
Married	62	55.9%
Single	44	39.6%
Widowed	2	1.8%
**Number of children**		
0	59	53.2%
1	13	11.7%
2	18	16.2%
3	7	6.3%
4	9	8.1%
5	5	4.5%
**Educational background**		
College	44	39.6%
Diploma	6	5.4%
High school	48	43.2%
Intermediate	9	8.1%
Elementary	4	3.6%
**Job position**		
Managers and Supervisors	28	25.2%
Workers	83	74.8%
**Smoking status**		
No	91	82.0%
Yes	20	18.0%
**Total working years in the factory**		
Less than one year	14	12.6%
1–3 years	23	20.7%
4–6 years	29	26.1%
7–9 years	33	29.7%
10 years and above	12	10.8%
**Sample size**	**111**	**100.0%**

*Bold value means P ≤ 0.05*.

### Safety Climate Perceptions

As recommended by NOSACQ-50, the reliability of each dimension of the study was tested separately, according to a measurable scale. Reliability was rated by computing Cronbach's alpha for all seven dimensions. Cronbach's alpha was between −0.3 to 0.6, which is considered poor. The negative value may show that correlations between items or factors are low or weak, possibly due to the small sample size. The reason for the limited sample size was the Covid-19 pandemic, which created difficulties in the data collection process, including meeting participants in industry settings.

In Table 3 the mean and standard deviation of each safety climate dimension are reflected. According to this scale reference, only one dimension received a good score, namely, peer safety communication, learning, and trust in co-workers. on the other hand, three dimensions, management safety empowerment, management safety justice, and safety competence, scored at a fairly low level in terms of workers trust in the efficacy of system safety. Three dimensions received significantly low scores of <2.70. These areas were management safety priority, commitment, and competence; workers safety commitment; and workers safety priority and risk non-acceptance.

**Table 3:** The mean perception of safety climate among the workers in the second industrial city – Dammam.

**Safety climate dimensions**	***M***	**SD**	**Cronbach's α**
1-Management safety priority, commitment, and competence.	2.654	2.053	0.076
2-Management safety empowerment.	2.921	2.243	0.282
3-Management safety justice.	2.874	1.795	−0.052
4-Workers' safety commitment.	2.620	1.533	−0.328
5-Workers' safety priority and risk non-acceptance.	2.314	2.621	0.292
6-Peer safety communication, learning, and trust in co-workers.	3.271	2.583	0.641
7-Safety competence, and workers' trust in the efficacy of system safety.	2.703	1.466	−0.109

### Occupational Health and Safety (OHS) Services

#### Occupational Health Services

In the occupational health services survey, we asked all participants if their company or factory had an occupational health service in the workplace. Of the respondents, 72.1% had no occupational health services in their company/factory. The remaining 27.9 % of participants reported that there was an occupational health service in their company/factory.

#### Medical Checkups

Regarding occupational health services, different medical checkups may be needed to ensure workers' health. The study's participants were asked whether their company/factory provided any medical checkups. The results showed that 70.3% received pre-placement medical checkups and 52.3% were given periodic medical checkups. However, only 2.7% had access to comprehensive health checkups and 18% of participants received no medical checkups from their company/factory.

### Occupational Hazard Exposure

Workers were exposed to different occupational hazards in various factories; however, noise reflected as the most common reported by 60.4%. Noise was followed by heat stress (40.5%), dust-related problems (30.6%), ventilation problems (23.4%), chemical exposure (20.7%), and vibration (19.8%). Radiation and lighting problems reflected as the lowest occupational hazards by 4.5% and 9.0% respectively.

### Gender Differences Gender Differences and Occupational Hazards

In Table 4 a chi-square test of independence was performed to examine the relationship between gender and occupational hazards. It showed a strong relationship between gender and participant responses to the question, do you hold a managerial position, e.g., manager, supervisor? calculated by the formula *X*^2^ (1, *N* = 111) = 16.173, *p* < 0.05, Phi = 0.382. Males were significantly more likely to be in a managerial position than females (73.3% to 26.7%). There was also a correlation between gender and responses to the question, do you work on a shift? based on the figures *X*^2^ (1, *N* = 111) = 27.335, *p* < 0.05, Phi = 0.496. Male worked on a shift schedule, and females did not (100–0%). Another finding was the relationship between gender and awareness of the term safety culture, *X*^2^ (1, *N* = 111) = 5.560, *p* = 0.018, Phi = 0.224. Males were significantly more likely to be aware of safety culture than females (53.7% to 46.3%). There was also a significant relationship between gender and responses to the question, How often do you use personal protective devices (PPE)? *X*^2^ (2, *N* = 111) = 7.558, *p* = 0.023, Phi = 0.261. Women were shown to be more regular users of PPE than men (64.4% to 35.6%). No significant correlations were found between gender and other categories on the questionnaire.

**Table 4:** Gender differences among workers regarding occupational hazards.

	**Female**	**Male**	**Chi-Square**	***P*-value**	**Phi**
**Do you have a managerial position, e.g. manager, supervisor?**					
No	56	25	16.173	0.000^*^	0.382
Yes	8	22			
**Do you work on a shift?**					
No	64	30	27.335	0.000^*^	0.496
Yes	0	17			
**Have you heard of the term “safety culture” before?**					
No	39	18	5.560	0.018^*^	0.224
Yes	25	29			
**Do you know what “safety culture” is?**					
No	46	29	1.280	0.258	0.107
Yes	18	18			
**Are you aware of the hazards associated with this job?**					
No	6	6	0.323	0.570	−0.054
Yes	58	41			
**Have you had formal training on hazards and safety measures to be taken?**					
No	17	9	0.830	0.362	0.086
Yes	47	38			
**Do you use personal protective devices (PPE)?**					
No	2	3	0.669	0.414	−0.078
Yes	62	44			
**How often do you use personal protective devices (PPE)?**					
Never	1	3	7.558	**0.023** ^*^	0.261
Occasionally	7	13			
Regularly	56	31			
**Have you had training at the workplace related to the occupational health and safety at workplace?**					
No	16	13	0.099	0.753	−0.030
Yes	48	34			
**Dose your company have occupational health & safety policy?**					
No	7	1	3.145	0.076	0.168
Yes	57	46			
**Does your company have an occupational Health Service in the Workplace?**					
No	42	38	3.121	0.077	−0.168
Yes	22	9			
**Pre-placement checkup**					
No	21	12	0.688	0.407	0.079
Yes	43	35			
**Periodic medical checkup**					
No	29	24	0.359	0.549	−0.057
Yes	35	23			
**Executive health checkup**					
No	63	45	0.747	0.387	082
Yes	1	2			
**No checkups**					
No	50	41	1.522	0.217	−0.117
Yes	14	6			
**Heat stress**					
No	32	34	5.611	0.018^*^	−0.225
Yes	32	13			
**Vibrations**					
No	54	35	1.674	0.196	0.123
Yes	10	12			
**Lighting**					
No	59	42	0.264	0.607	0.049
Yes	5	5			
**Radiations**					
No	62	44	0.669	0.414	0.078
Yes	2	3			
**Ventilation problems**					
No	47	38	0.830	0.362	−0.086
Yes	17	9			
**Dust related problems**					
No	51	26	7.573	0.006^*^	0.261
Yes	13	21			
**Chemical exposure**					
No	57	31	8.807	0.003^*^	0.282
Yes	7	16			
**No exposed**					
No	52	35	0.735	0.391	0.081
Yes	12	12			
**Ergonomic**					
No	28	37	13.659	0.000^*^	−0.351
Yes	36	10			
**Dermatitis**					
No	58	46	2.409	0.121	−0.147
Yes	6	1			
**Respiratory problems**					
No	54	44	2.239	0.135	−0.142
Yes	10	3			
**Hematological problems**					
No	63	45	0.747	0.387	0.082
Yes	1	2			
**Renal diseases**					
No	64	46	1.374	0.241	0.111
Yes	0	1			
**Liver diseases**					
No	64	47			
Yes	0	0			
**Central nerve system**					
No	61	47	2.264	0.132	−0.143
Yes	3	0			
**Cardiovascular diseases**					
No	64	47			
Yes	0	0			
**Stress**					
No	49	36	0.000	0.997	0.000
Yes	15	11			
**Cancers**					
No	64	47			
Yes	0	0			
**No occupational health problems**					
No	44	19	8.858	**0.003** ^*^	0.282
Yes	20	28			

* *Bold value means P ≤ 0.05*.

A chi-square test of independence was performed to examine the relationship between gender and OHS services provided to the factory workers. There was no significant relationship between gender and responses to the question, does your company have an occupational health service in the workplace? *X*^2^ (1, *N* = 111) = 3.121, *p* = 0.077, Phi = −0.168. There was also no significant relationship between gender and the categories of pre-placement checkup, periodic medical checkup, executive health checkup, and had no checkups.

A chi-square test of independence was performed to examine the relationship between gender and exposure to occupational health risks. There was a significant relationship between gender and heat stress, dust exposure, and chemical exposure. Women were found to be more likely to be exposed to heat stress than men (71.1–28.9%), while men were more likely to experience dust-related problems than women (61.8–38.2%). Males were also more likely to be exposed to chemical than females (69.6–30.4%).

A chi-square test of independence was performed to examine the relationship between gender and occupational health problems. There was a significant relationship between gender and occupational health problems in terms of ergonomics, *X*^2^ (1, *N* = 111) =13.659, p = 0.000, p < 0.05, Phi = −0.351. Women were found to be more susceptible to ergonomic problems than men (78.3–21.7%). Additionally, there was a correlation between gender and participants who had experienced no occupational health problems, *X*^2^ (1, *N* = 111) = 8.858, *p* = 0.003, *p* < 0.05, Phi = 0.282. The results reflected that 58.3% of male participants had not experienced occupational health problems, compared to 41.7% of female respondents. The remaining categories showed no significant gender differences.

### Gender Differences and Safety Climate

In the sample dataset, workers reported their perceptions of the safety climate, and the information was analyzed to determine whether any gender differences existed. This involved testing whether the sample means rank for males and females were statistically different by using Mann Whitney test.

A significant difference was found in the safety priority and risk non-acceptance dimension and workers' safety commitment between female and male workers' perceptions. Female have shown more positive toward safety and precaution measurement than men = 0.05. There were no significant differences between men and women in any of the other dimensions as shown in Table 5.

**Table 5:** Gender differences among workers regarding safety climate perception.

**Dimensions**	**N**	**Mean rank**	**Mann-Whitney**	**Significant level**
**1-Management safety priority, commitment, and competence**.
Female	64	59.37	1,288.50	0.191
Male	47	51.41		
**2-Management safety empowerment**.
Female	64	54.36	1,399.00	0.526
Male	47	58.23		
**3-Management safety justice**.
Female	64	51.15	1,193.50	0.058
Male	47	62.61		
**4-Workers' safety commitment**.
Female	64	61.09	1,178.50	0.041^*^
Male	47	49.07		
**5-Workers' safety priority and risk non-acceptance**.
Female	64	62.77	1,070.50	0.009^*^
Male	47	46.78		
**6-Peer safety communication, learning, and trust in co-workers**.
Female	64	52.95	1,308.50	0.239
Male	47	60.16		
**7-Safety competence, and workers' trust in the efficacy of system safety**.
Female	64	55.34	1,461.50	0.786
Male	47	56.90		

* *Bold value means P ≤ 0.05*.

## Discussion

This study assessed safety climate perceptions among workers, identified different occupational health hazards and services, and compared the perspectives of genders on occupational safety climates and hazards in the industrial sector of Dammam, Saudi Arabia.

The dimension of workers safety priority and risk non-acceptance had the lowest score overall, which indicates a low level of safety with a great need for improvement. This finding was also reported by Marín et al. across 353 workers (11). In contrast, while peer safety communication, learning, and trust in co-workers scored highest in this study, other studies found that workers trust in the efficacy of system safety and workers safety commitment scored highest (11, 12). When comparing the results to an international NOSACQ-50 database of 57,270 worker respondents around the world, the dimensions of management safety empowerment and management safety justice had similarly low scores, pointing to a need for improvement. However, the scores of other five dimensions were contrary to our findings (13). These differences in the results may be attributed to the stratified small sample selection size in this study, while the NOSACQ-50 international database was compiled from volunteer participants recruited by companies with vested interests.

As Kines et al. (14) stated based on the designed theoretical framework, a score of 3.00–3.30 points to a fairly good safety climate with a slight need for improvement a score of 2.70–2.99 indicates a relatively low safety climate with the need for improvement, and a score below 2.70 reveals a poor safety climate with a great need for improvement.

In all five industries, noise was the most common occupational hazard, reported by 60.4% of study subject. In accordance with the present results, a previous study reported that noise was one of the most frequent occupational hazards reported by 78.3% (15). Additionally, 34% of the factories and 27.4% of the participants respectively produced a high level of noise (16, 17).

The second most common occupational hazard exposure was heat stress, reported by 40.5% of the present study participants. This result may be explained by the fact that most workers who reported exposure to heat stress work in contact with or close to a machine or material that produces heat on the production line. Based on the literature, studies found that heat stress was the second-highest physical hazard reported by 22.8% (17), and the third-highest hazard reported by 65.2% (15).

Occupational health services help to prevent illness, injury, or even death in the workplace. OHS aims to promote and maintain physical and mental health. The findings of this study showed that 72.1% of participants did not have access to any occupational health services in their workplace. This is despite the emphasis that many authoritative bodies, including the ILO and WHO, have prioritized on the need for occupational health services in recent years. These can contribute to the country's economic development by improving productivity, product quality, work motivation, and job satisfaction. Additionally, OHS can contribute to creating better quality of life for working people and the community (18).

MLSD (19) advises that workers who are at risk of developing an occupational disease should be examined by a physician at least once a year. Even though the factories in this study were seeking to comply with these regulations, it was revealed that only 52.3% of the participants had periodic medical checkups, while 70.3% of the participants had pre-placement medical checkups and 18% had none.

The Occupational Safety and Health Administration (1) in the united states suggests that in case of absence of a clinic, hospital, or physician that is reasonably accessible to injured workers in terms of time and distance from the worksite, an individual who has a valid certificate in first aid shall be available at the worksite to render basic treatment. This study discovered that only one of five factories did not have a dedicated OHS officer.

The last question in the survey was, are there any gender differences among workers regarding occupational safety climate and hazards? The only safety climate dimension that indicated a statistically significant difference between men and women was workers safety priority and risk non-acceptance. However, there is a deficit in the number of reviewed studies comparing gender regarding the safety climate in the industrial sector.

In Saudi Arabia, MLSD (19) establishes workplaces policies especially for women to ensure a safe work environment. For example, inhibit females from working in high-risk tasks policies, working hours policies, pregnancy, and labor period policies. Implementing these regulations contributes to enhancing occupation and health safety.

In the other hand, a case study in Hong Kong examined the relationship between safety climate and personal characteristics (20). They found that there was no influence of gender in the perception of safety climate. However, they, reported that 95% of their respondents were male, which may explain their finding (20).

Surprisingly, gender differences in hazard exposure were statistically significant. Males reported that more likely to be exposed to chemicals and dust, while females were more prone to heat stress. The reason is the different type of jobs worked by men and women in Saudi Arabia. As per Saudi labor law, females reported that they are more likely to work in low-risk industries such as food processing, while males are more likely to work in environments that present medium to high risks such as mining or oil and gas extraction or processing. This means workers are exposed to different occupational hazards depending the type of job and industry. There was also a strong relationship between gender differences and ergonomic health problems, with women more likely to experience ergonomic problems than men. This study supports evidence from previous research (9, 21). Some of these findings relate specifically to the type of job and related health risks. Companies typically hire females to work in low- to medium-risk industries that commonly require them to perform repetitive motion tasks. Therefore, this type of work may result in ergonomic health problems.

## Conclusion

This study concludes that there is a significant difference between females and males in the areas of workers' safety priority and risk non-acceptance. The research has also shown that 18% of the participants did not receive any medical checkups from their company/factory. Another major finding in this study was that noise was the most common occupational hazard by 60.2%. Additionally, ergonomic problems were shown to be more likely to affect females than males. The main weakness of this study was its small sample size, which did not allow the results to be generalized to the second industrial city population. This research obstructs the target sample size due to the covid-19 pandemic and voluntary survey response. Moreover, recall bias was one of the weaknesses in this study because the questionnaire included some questions that focused on previous events. Notwithstanding the relatively limited sample, this work offers valuable insights into gender differences because 57.7% of participants were female, compared to many other studies that only represented males. In light of these results, the authors of this study recommend that industries follow MLSD rules, including providing training for all workers, especially new employees; providing occupational health services; and keeping records of the results of workers' annual medical checkups. Further studies on the current topic involving a greater sample size would be a great help in generalizing the result.

## Data Availability Statement

The original contributions presented in the study are included in the article/supplementary material, further inquiries can be directed to the corresponding author.

## Author Contributions

All authors listed have made a substantial, direct, and intellectual contribution to the work and approved it for publication.

## Conflict of Interest

The authors declare that the research was conducted in the absence of any commercial or financial relationships that could be construed as a potential conflict of interest.

## Publisher's Note

All claims expressed in this article are solely those of the authors and do not necessarily represent those of their affiliated organizations, or those of the publisher, the editors and the reviewers. Any product that may be evaluated in this article, or claim that may be made by its manufacturer, is not guaranteed or endorsed by the publisher.
